# Lactic Acid Bacteria and Yeast Fermentation to Improve the Nutritional Value of *Ulva rigida*

**DOI:** 10.3390/md23030106

**Published:** 2025-02-28

**Authors:** Marta Brandão, Diogo J. Marques, Sofia Sousa, Marília Mateus, Helena M. Pinheiro, M. Manuela R. da Fonseca, Carla Pires, Maria Leonor Nunes, António Marques, M. Teresa Cesário

**Affiliations:** 1 iBB—Institute for Bioengineering and Biosciences, Bioengineering Department, Instituto Superior Técnico, Universidade de Lisboa, 1049-001 Lisboa, Portugal; martabrandao@tecnico.ulisboa.pt (M.B.); diogo.j.marques@tecnico.ulisboa.pt (D.J.M.); sofia.s.sousa@tecnico.ulisboa.pt (S.S.); helena.pinheiro@tecnico.ulisboa.pt (H.M.P.); manuela.fonseca@tecnico.ulisboa.pt (M.M.R.d.F.); 2 Associate Laboratory i4HB—Institute for Health and Bioeconomy, Instituto Superior Técnico, Universidade de Lisboa, 1049-001 Lisboa, Portugal; 3 Division of Aquaculture, Upgrading and Bioprospection (DivAV), Portuguese Institute for the Sea and Atmosphere (IPMA IP), 1749-077 Lisboa, Portugal; cpires@ipma.pt (C.P.); amarques@ipma.pt (A.M.); 4 CIIMAR, Interdisciplinary Center of Marine and Environmental Research, University of Porto, 4450-208 Matosinhos, Portugal; lnunes@ciimar.up.pt

**Keywords:** macroalgae, *Ulva rigida*, lactic acid fermentation, ethanol fermentation, fermented seaweed, single cell protein, bioactivity, bioaccessibility

## Abstract

Aquaculture reliance on fishmeal protein has become a bottleneck due to long-term sustainability concerns and increasing costs. Given its abundance and nutrient-rich profile, the green macroalga *Ulva rigida* is a promising alternative protein source. However, the bioaccessibility of its proteins is hindered by an embedding matrix of ulvan, a gel-forming polysaccharide. Saccharification of the alga crude fiber followed by microbial fermentation improves protein bioaccessibility and leads to products of higher protein content and quality. Also, upon fermentation, the nutritional and bioactive properties of these feed ingredients are enhanced, since microorganisms synthesize vitamins, new proteins, and essential amino acids. The carbohydrate fraction of *Ulva rigida* was hydrolyzed into a sugar-rich syrup and subsequently used as a substrate in microbial fermentations. Three types of fermentation were tested, namely, with a consortium of four lactic acid bacteria (LAB), with *Saccharomyces cerevisiae*, and with a co-culture of lactobacilli and yeast. A functional analysis of lyophilized whole-fermentation broths revealed that the yeast-fermented products had stronger antioxidant properties when compared to the LAB-fermented products. The protein bioaccessibility in the fermented products was 11- to 12-fold higher than that of the raw alga. These findings highlight the potential of utilizing *S. cerevisiae* and lactobacilli starter cultures in seaweed fermentation to produce *Ulva*-based feed ingredients.

## 1. Introduction

To address a global protein-shortage scenario, a steady development in food technologies tackling alternative protein sources is underway. This opens the possibility for seaweed utilization. Macroalgae, commonly known as seaweeds, are regarded as nutrient-rich food sources because they contain substantial amounts of proteins, carbohydrates, minerals, and fibers while being relatively poor in lipids (Yong et al., 2022) [1]. This nutritional profile makes them desirable for inclusion in balanced diets. Furthermore, macroalgae offer additional benefits such as high biomass productivity that far exceeds that of terrestrial biomass and no agricultural input of fertilizers as they grow naturally in the marine environment. Consequently, their cultivation does not occupy fertile soil or freshwater, both increasingly scarce resources (Fernandes et al., 2019) [2]. Thus, this marine resource has the potential to become a crucial feedstock for food and feed applications.

Among the different classes of proteins identified up to date in seaweed, it is important to emphasize glycoproteins, lectins, and phycobiliproteins. Lectins are a group of glycoproteins that exhibit antibacterial, antiviral, anticancer, anti-inflammatory, and anti-HIV properties (Pangestuti and Kim, 2015) [3], while phycobiliproteins have received great attention due to their numerous medicinal uses, e.g., as antioxidant, antiangiogenic, and neuroprotective agents (Cuellar-Bermudez et al., 2015) [4]. Proteins identified in *Ulva* sp. belong to the group of glycoproteins that are carbohydrate-binding proteins [5,6]. Glycoproteins are found in the cell wall and on cell surfaces, and it is hypothesized that they have a key role in the physiological functions of seaweed cell walls [5]. Very recently, arabinogalactan proteins (AGPs), like glycoproteins, were isolated for the first time from *Ulva lactuca* (Ulvophyceae) (Přerovská et al., 2021) [7]. AGPs are proteoglycans consisting of two distinct moieties, the carbohydrate and the protein domains, which can vary in structure and composition. In the case of Ulva sp., arabinose and galactose were not the most prevalent monosaccharides, but instead 3-O-methyl-hexose, which has never been described in the AGPs. Protein nutritional value is determined by two main parameters, namely bioaccessibility, digestibility, and amino acid profile (Demarco et al., 2022) [8]. Regrettably, despite possessing rich amino acid profiles, seaweeds show poor protein digestibility in their raw and unprocessed form because their proteins are embedded in complex structures of algal polysaccharides. These indigestible fibers resist the action of digestive enzymes, resulting in low bioaccessibility. Therefore, to improve bioaccessibility, processes such as saccharification, proteolysis, and fermentation have been applied, which deconstruct those complex structures, releasing smaller molecules, such as peptides and simpler sugars, that can be further used as substrates in microbial fermentations (Reboleira et al., 2021) [9]. Additional benefits arise from seaweed fermentation because microorganisms enrich the algal material with vitamins, essential amino acids, and fatty acids, enhancing its nutritive value (Gänzle, 2015) [10]. When compared to lignocellulosic biomass, seaweed polysaccharides can be hydrolyzed under milder conditions due to the absence of lignin. Acid hydrolysis efficiently produces sugar-rich syrups but often generates compounds that can inhibit fermentation, such as the byproducts of sugar degradation: hydroxymethylfurfural (HMF) and furfural. The latter are known to be detrimental to microbial growth and metabolite production, decreasing fermentation yields (Giacon et al., 2022) [11]. To prevent or reduce inhibitor production, enzymatic hydrolysis of the algal polysaccharides would be an alternative. However, while cellulases and amylases are easily available, there is a lack of commercial bulk enzymes able to saccharify complex algal polysaccharides. The remaining alternatives are, thus, either to use microorganisms able to saccharify these complex polysaccharides and grow on the produced simpler sugars or to chemically hydrolyze the polymers to simple sugars and subsequently use fermenting microorganisms that are tolerant to the inhibitors released during acid hydrolysis.

**Figure 1:**
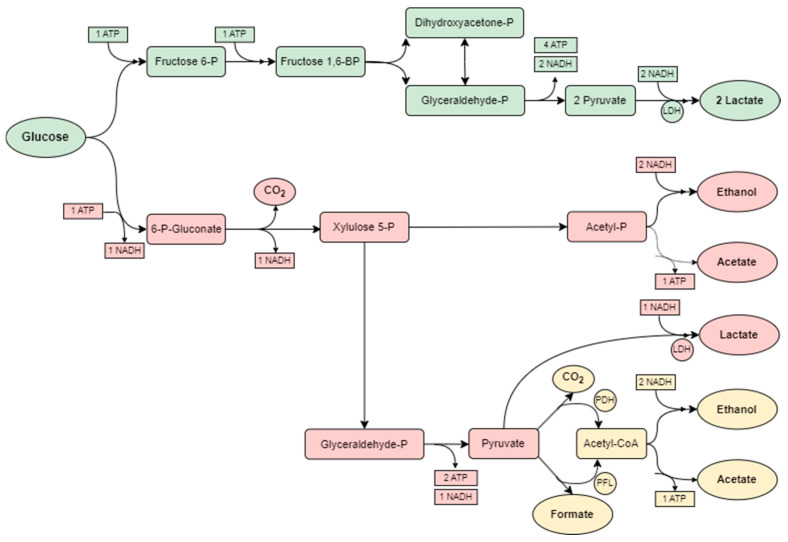
Metabolic pathways of LAB species for the synthesis of LA: homofermentative (green), heterofermentative (red), and mixed acid fermentation (yellow). Legend: LDH—lactate dehydrogenase; PFL—pyruvate formase lyase; PDH—pyruvate dehydrogenase; BP—biphosphate. Adapted from Hofvendahl and Hahn-Hägerdal, 2000 [15].

Lactic acid and ethanol fermentations are well-established core processes in the beverage and food sectors, as they produce a broad range of aromas and flavors that are extremely difficult to replicate through alternative means while ensuring product safety. Traditionally, lactic acid-producing bacteria (LAB) have been widely employed in fermentation processes due to their ability to convert carbohydrate substrates into lactic acid (LA) as the main end-product. Moreover, these bacteria possess metabolic routes to synthesize products such as short-chain fatty acids, amines, vitamins, and exopolysaccharides (Y. Wang et al., 2021) [12]. A large number of LAB strains are “Generally Recognized as Safe” (GRAS) according to the United States Food and Drug Administration (FDA). Furthermore, many have also been granted the status of “Qualified Presumption of Safety” (QPS) by the European Food Safety Authority (EFSA), including the genera *Lactococcus*, *Carnobacterium*, *Oenococcus*, *Leuconostoc*, *Streptococcus*, *Pediococcus*, and the former *Lactobacillus* (Barcenilla et al., 2022) [13]. LAB are capable of metabolizing glucose through three different pathways, known as obligatory heterofermentative, obligatory homofermentative, and facultative heterofermentative (Figure 1). The obligatory heterofermentative strains decompose glucose via the pentose–phosphoketolase pathway, resulting in ethanol, acetic acid, and carbon dioxide being synthesized in equimolar amounts, in addition to LA. Heterofermentative bacteria include *Lactobacillus pentosus*, *Lactobacillus bifermentans*, and *Levilactobacillus brevis* (formerly *Lactobacillus brevis*), to name a few. In the homofermentative cascade, glucose is metabolized via the Embden–Meyerhof–Parnas (EMP) pathway (glycolysis), so bacteria in this group are able to convert hexoses to LA but do not hold the capacity to degrade gluconate or pentoses. As a direct consequence, LA is the only product formed, and thus, homofermentative strains are used for the commercial production of this organic acid. These include *Lactobacillus delbrueckii*, *Lactococcus lactis*, *Lactobacillus casei*, and *Lactobacillus acidophilus* (Gänzle, 2015) [10]. The facultative heterofermentative pathway (or mixed acid fermentation) may occur under conditions in which some lactic acid bacteria regarded as homofermentative use the pentose phosphate pathway to metabolize certain substrates. In this sense, a mixed fermentation may be conducted in which both pathways can occur in an alternate fashion, depending on environmental cues like temperature, pH, or nutrient availability (Pot et al., 2009) [14].

**Figure 2:**
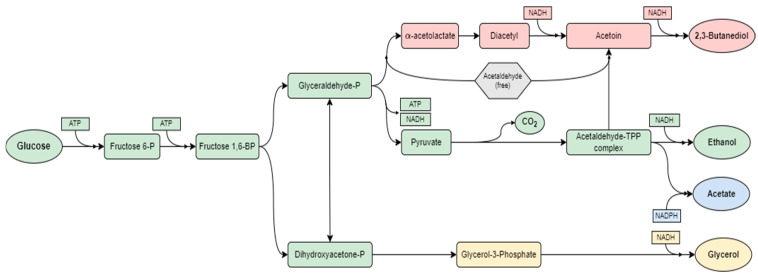
Ethanol biosynthetic routes in *Saccharomyces cerevisiae*. The biochemical process wherein glucose is enzymatically converted to pyruvate is known as glycolysis. Subsequently, pyruvate undergoes decarboxylation, leading to the formation of acetaldehyde. The latter is then reduced by NADH, resulting in ethanol (green). Secondary fermentation metabolites encompass glycerol (yellow), acetate (blue), 2–3 butanediol, and acetoin (red), which collectively act as flavor precursors. Adapted from Macedo and Brigham, 2014 [20].

Yeasts are unicellular ubiquitous eukaryotic fungi that are commonly found in ripe fruits, vegetables, and other plant materials (Fernandes et al., 2022; Maicas, 2020) [16,17]. These microorganisms play a crucial role in food fermentation by imparting distinctive flavors, textures, and aromas through secondary metabolic routes, thereby modifying the sensory properties of foods. *Saccharomyces cerevisiae* (Fungi, Saccharomycetes) is the prevalent strain in ethanol fermentation due to its high ethanol production yield, rapid sugar consumption, fast growth, and aptitude to thrive in harsh environmental conditions, including high ethanol and organic acid concentrations, low pH values (3.0–3.5), and nutrient scarcity (Albergaria et al., 2016) [18]. *S. cerevisiae* is a mesophilic yeast that exhibits ethanol production within the temperature range of 20 °C to 35 °C, with an optimum at 30 °C. Regarding pH, the optimum values fall between 4.0 to 5.0, with lower values resulting in longer incubation periods without a significant impact on ethanol concentration, and higher values considerably reducing ethanol production (Mohd Azhar et al., 2017) [17,19]. Under anoxic conditions, *S. cerevisiae* converts sugars into ethanol and carbon dioxide through a series of enzymatic reactions, generating metabolic energy (Maicas, 2020) [17]. Under certain conditions, the carbon flux may be redirected to produce small quantities of 2,3-butanediol, acetoin, and acetate, which contribute to the flavor characteristics of alcoholic beverages (Figure 2). Additionally, glycerol is formed from dihydroxyacetone–phosphate. Glycerol is an important constituent of wine, as it enriches its sensory profile with sweetness, fullness, and smoothness (Macedo and Brigham, 2014) [20].

In addition to generating fermentation metabolites, the growth of the fermentative strains increases the protein content of the whole biomass slurry, originating a protein-rich fermented product. In addition, lactic acid bacteria are probiotics that provide health benefits by modifying the host-associated microbial community (Hasler 2002) [21]. Despite the potential of fermented seaweed for food and feed purposes, fermentation results from these biomass resources are scarce in the literature. Table 1 summarizes the results of published studies using seaweed as fermentation substrates and different lactic acid bacteria and yeast species aiming at the production of fermented seaweed food products and feedstocks for aquaculture.

The present work aims to establish an optimized and scalable method for efficient saccharification of the carbohydrate fraction of *Ulva rigida*. In addition, the subsequent fermentation conditions, using a mixed consortium of four lactobacilli (4LAB) and/or *Saccharomyces cerevisiae*, are tuned to improve lactic acid and ethanol titers and increase the total protein content of the biomass, as well as its bioaccessibility. *Ulva rigida* was chosen as an algal feedstock due to its availability and high carbohydrate content.

## 2. Results

### 2.1. Total Carbohydrate Quantification

Different batches of *Ulva rigida* produced in controlled cultivation systems and received in the form of dried flakes were analyzed for their carbohydrate content using the NREL protocol of total carbohydrate hydrolysis.

Among the monosaccharides and per dry weight of original biomass (dw), glucose was dominating (0.21 to 0.31 g/g dw), rhamnose was the second largest component (0.07 to 0.11 g/g dw), and xylose held a small contribution in the total carbohydrate content (0.03 to 0.09 g/g dw). All three monomers were monitored in the subsequent saccharification and fermentation assays.

### 2.2. Acid Hydrolysis for Ulva Saccharification

The release of sugars from *Ulva* polysaccharides was evaluated using dilute acid hydrolysis with HCl and H_2_SO_4_ at different concentrations. Preliminary assays at 1% and 3% (*w*/*v*) gave good results in terms of carbohydrate hydrolysis, prompting the concentration range selected for this study.

**Figure 3:**
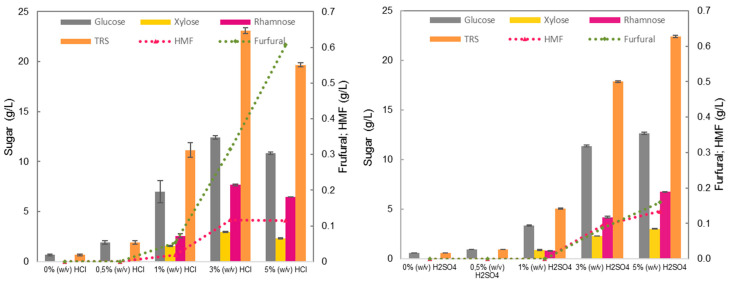
*U. rigida* dilute acid hydrolysis with 0.5, 1, 3, and 5% (*w*/*v*) H_2_SO_4_ (**left**) and HCl (**right**), for 30 min at 121 °C. Total released sugars (TRS) is the sum of glucose, xylose, and rhamnose concentrations.

As shown in Figure 3, the hydrolysis of complex carbohydrates in *Ulva* biomass into monosaccharides is influenced by the type and concentration of the acid agent. With sulfuric acid, all monosaccharides reached the highest concentrations at 5% (*w*/*v*), while with hydrochloric acid, the best performance was achieved at 3% (*w*/*v*). The titers of the inhibitors furfural and HMF were consistently higher in the HCl treatment. For this reason, further processing of the latter hydrolysates was not considered, and treatment with sulfuric acid was adopted. Because a significant increase in furfural and HMF concentrations was registered from 3% (*w*/*v*) to 5% (*w*/*v*) H_2_SO_4_, only the pretreatments with 1% (*w*/*v*) and 3% (*w*/*v*) H_2_SO_4_ were tested for subsequent enzymatic hydrolysis.

### 2.3. Enzymatic Hydrolysis of Ulva Cellulose

**Figure 4:**
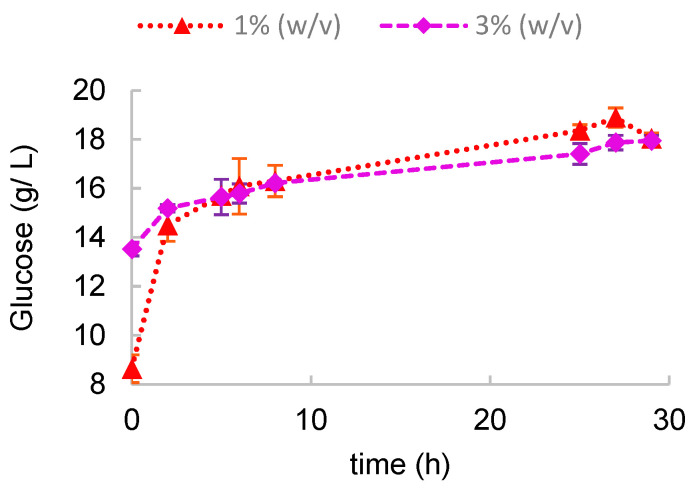
Enzymatic hydrolysis at 50 °C and pH 4.8 during 30 h of *Ulva* suspensions pre-treated with dilute H_2_SO_4_ at 1 and 3% (*w*/*v*).

To further improve the hydrolysis of the cellulose fraction in *Ulva* biomass after the acid pre-treatment with 1% and 3% H_2_SO_4_, a cellulolytic enzyme cocktail composed of cellulases and β-glucosidase was used. Cellulases hydrolyze the β-(1→4) glycosidic bonds in cellulose molecules (Cavaco-Paulo, 1998) [28], and β-glucosidase complements this process with the hydrolysis of cellobiose to glucose (Keller et al., 2020) [29]. The results are depicted in Figure 4.

The initial glucose concentration was higher in the hydrolysate from previous hydrolysis with 3% (*w*/*v*) H_2_SO_4_. However, beyond the fifth hour of enzyme treatment, the glucose concentrations in both flasks were very similar and increased only slightly thereafter, attaining nearly 20 g/L. This corresponds to overall hydrolytic yield values of 0.63 g _released glucose_/g _glucose in *Ulva*_ and 0.60 g _released glucose_/g _glucose in *Ulva*_ for the 1% (*w*/*v*) and 3% (*w*/*v*) H_2_SO_4_ pre-treatments, respectively.

### 2.4. Optimum Ulva Pre-Treatment for Microbial Growth

**Figure 5:**
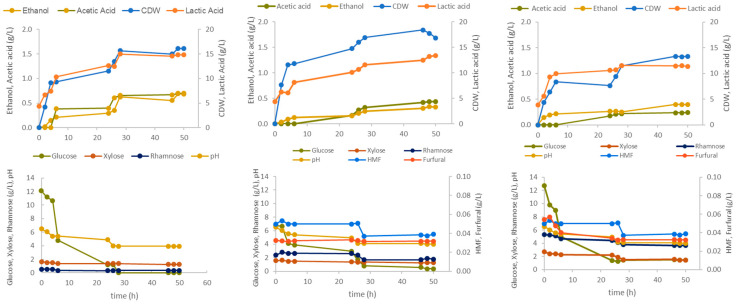
Time course of the 4LAB lactobacilli consortium fermentations using *Ulva* hydrolysates from different pre-treatments: 1% H_2_SO_4_ + cellulases (**left**), 3% H_2_SO_4_ (**middle**), and 5% H_2_SO_4_ (**right**). The hydrolysates were used as main carbon source, supplemented with corn steep liquor (CSL) and minerals.

**Figure 6:**
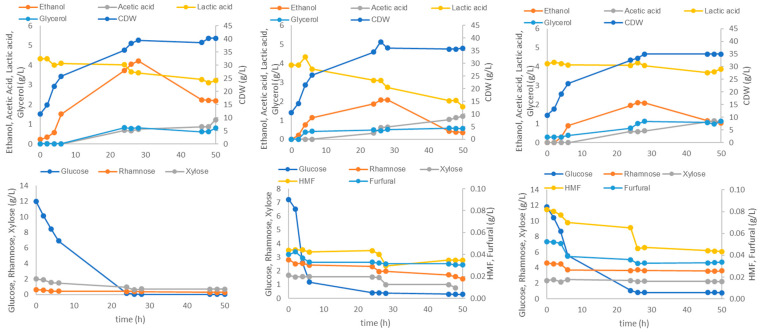
Time course of the *S. cerevisiae* fermentations using *Ulva* hydrolysates from different pre-treatments: 1% H_2_SO_4_ + cellulases (**left**), 3% H_2_SO_4_ (**middle**), and 5% H_2_SO_4_ (**right**). The hydrolysates were used as the main carbon source, supplemented with CSL and minerals.

Based on the obtained results, different pre-treatment strategies of *Ulva* biomass were identified as promising for its subsequent use in microbial fermentation. *Ulva* treated with 3% H_2_SO_4_ exhibited a monosaccharide profile similar to that of the 5% H_2_SO_4_ product, except for rhamnose, but lower levels of furfural and HMF, and enzymatic treatment of *Ulva* pre-treated with 1% H_2_SO_4_ efficiently released glucose. The hydrolysates from these three different pre-treatments (1% H_2_SO_4_ + cellulases, 3% H_2_SO_4_, and 5% H_2_SO_4_) were tested as carbon substrates for the growth of either the 4LAB consortium or *S. cerevisiae* in shake flask assays. The results are given in Figure 5 and Figure 6, respectively.

As observed in Figure 5, the lactobacilli consortium produced not only lactic acid but also acetic acid and ethanol, demonstrating the heterofermentative nature of some of the species. In all tested conditions, lactic acid rapidly accumulated within the first 10 h and reached a plateau at 28 h. The sharp drop in medium pH from 5.0 (at 26 h) to 4.0 (at 28 h) is probably what caused lactic acid and cell concentration levels to stabilize. As pH decreases due to lactic acid accumulation, its undissociated form increases, which is responsible for cytoplasm acidification and the failure of proton driving forces, causing LAB growth inhibition (Othman et al., 2017) [30].

Regarding sugar uptake, the glucose was completely consumed at 48 h in the 1% H_2_SO_4_ + cellulases medium, while residual glucose levels were still measured in the assays with the hydrolysates from 3% H_2_SO_4_ (0.6 g/L) and 5% H_2_SO_4_ (1.45 g/L). Xylose and rhamnose were barely uptaken from the 1% H_2_SO_4_ + cellulases hydrolysate, while in the 3% H_2_SO_4_ assay, there was some consumption of rhamnose, but little of xylose. And in the 5% H_2_SO_4_ assay, both sugars were consumed. Cell growth was followed by measuring cell dry weight (CDW), and all values were corrected for the initial medium levels due to the suspended matter present in the hydrolysates. The final 4LAB CDW values were similar for all hydrolysates.

A similar study was carried out with *S. cerevisiae* (Figure 6). The ethanol concentration reached its peak at 28 h, after which it decreased until the end of the assay. The onset of this decline in ethanol levels coincided with the depletion of glucose. An inhibitory effect was only observed in the hydrolysate prepared with 5% H_2_SO_4_. The lactic acid detected in this assay comes from the corn steep liquor used in medium formulation (cf. 4.6), where it is present at up to 10–30% of the corn extractives on a dry basis (Loy and Lundy, 2019) [31]. While the synthesis of acetic acid was comparable across all assays, the glycerol concentrations attained higher values in those using the harsher chemical pre-treatment.

In general, all of the selected microorganisms were able to grow on the *Ulva* hydrolysate-based media, despite the presence of inhibitors in those from the harsher hydrolysis settings. To overcome the reduction of medium pH during cultivation, assays were thereafter conducted in a bench-scale bioreactor with automatic pH control. The *Ulva* hydrolysate produced using the 3% H_2_SO_4_ pre-treatment was selected as a substrate for lactobacillus and yeast cultivations.

### 2.5. Fermentation Scale-Up

Three different bench-scale submerged fermentations were carried out in a 2 L stirred-tank bioreactor (STR) using *Ulva* hydrolysate (3% H_2_SO_4_) as the main carbon source and three different inocula, namely, the *Lactobacillus* consortium (4LAB), a co-culture of 4LAB and yeast (*S. cerevisiae*), or *S. cerevisiae* alone.

The hydrolysate was supplemented with CSL as an organic nitrogen source (CSL is rich in vitamins, lactic acid, and proteins) (Zhou et al., 2022) [32] and a mineral medium containing di-ammonium hydrogen citrate as an inorganic nitrogen source. This medium with CSL was used, instead of the MRS medium commonly employed for LAB growth, to reduce production costs and potentially improve product bioactive properties. To further increase the nitrogen supply and, hence, foster biomass and protein content, ammonium hydroxide was used for pH control. Fed-batch cultivations were carried out with the supplemented hydrolysate in the initial batch phase, followed by a fed-batch phase with a concentrated glucose solution as the feed. Glucose feed pulses were added manually at regular time intervals to avoid sugar exhaustion. The fermentations ended when the concentrations of lactic acid and/or ethanol stabilized.

#### 2.5.1. *Lactobacillus* Fermentation

**Figure 7:**
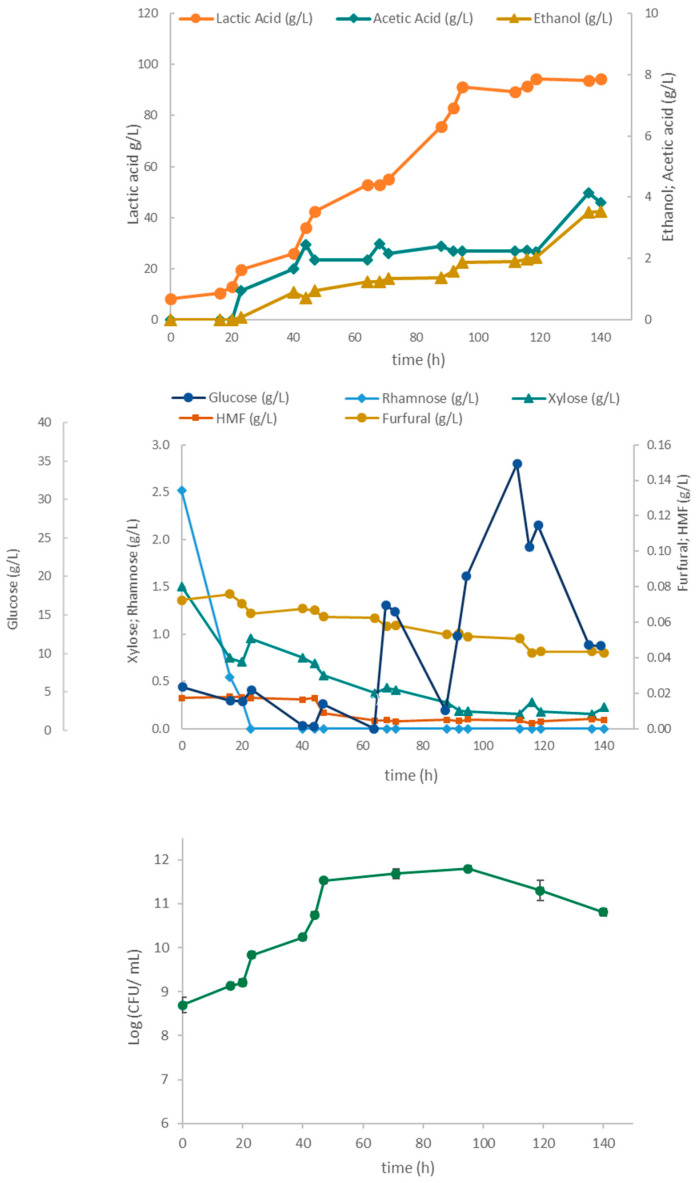
Time course of the 4LAB consortium cultivation in a 2 L stirred tank bioreactor using *Ulva* hydrolysate in the batch phase (up to 20 h) followed by a fed-batch phase with a concentrated glucose solution as feed (pulses roughly every 20 h).

Figure 7 shows the sugar consumption and metabolite production in fermentation using the LAB consortium. During the first 16 h of fermentation, both glucose consumption and metabolite production were minimal, and cell growth was slow. This lag phase may have been caused by the presence of the toxic compounds HMF and furfural in the *Ulva* hydrolysate, given that the LAB inocula were prepared on an MRS medium instead of using seaweed hydrolysate to promote acclimatization. Nonetheless, the LAB consortium was able to lower inhibitory compound concentrations in the broth, even reducing the furfural to negligible levels. The rapid consumption of xylose and rhamnose during the first hours of fermentation could be explained by low glucose availability (3.9 g/L), thus alleviating carbon catabolite repression, resulting in the consumption of other sugars and even in the depletion of rhamnose after 23 h. At 95 h, both the biomass (6.3 × 10^11^ CFU/mL) and the lactic acid (91.4 g/L) concentrations had nearly attained their maxima, while at 136 h, acetic acid and ethanol reached titers of 4.2 g/L and 3.5 g/L, respectively. To avoid the interference of *Ulva* solids in cell dry-weight measurements, LAB growth was followed by counting the colony-forming units (CFU). CFU values experienced a rapid increase up to 47 h, followed by growth deceleration until 95 h, before declining towards the end of the fermentation. The exponential and decelerating cell growth phases were associated with lactic acid production.

#### 2.5.2. Yeast and *Lactobacilli* Mixed Fermentation

Based on the indications from previous assays (unpublished data), a sequential fermentation with yeast and then LAB was chosen to take advantage of both species’ metabolic activities for the co-fermentation of *Ulva* hydrolysate. Because LAB can multiply in the presence of high ethanol concentrations, it was decided to introduce the yeast inoculum first and add the 4LAB inoculum later in the fermentation. Specifically, *Levilactobacillus brevis*, *Lactobacillus casei*, *Lactobacillus plantarum*, and *Lacticaseibacillus rhamnosus* (the species used in the 4LAB consortium) are known to grow under ethanol concentrations up to 8, 15, 12, and 12% (*v*/*v*), respectively (Pittet et al., 2011) [33].

**Figure 8:**
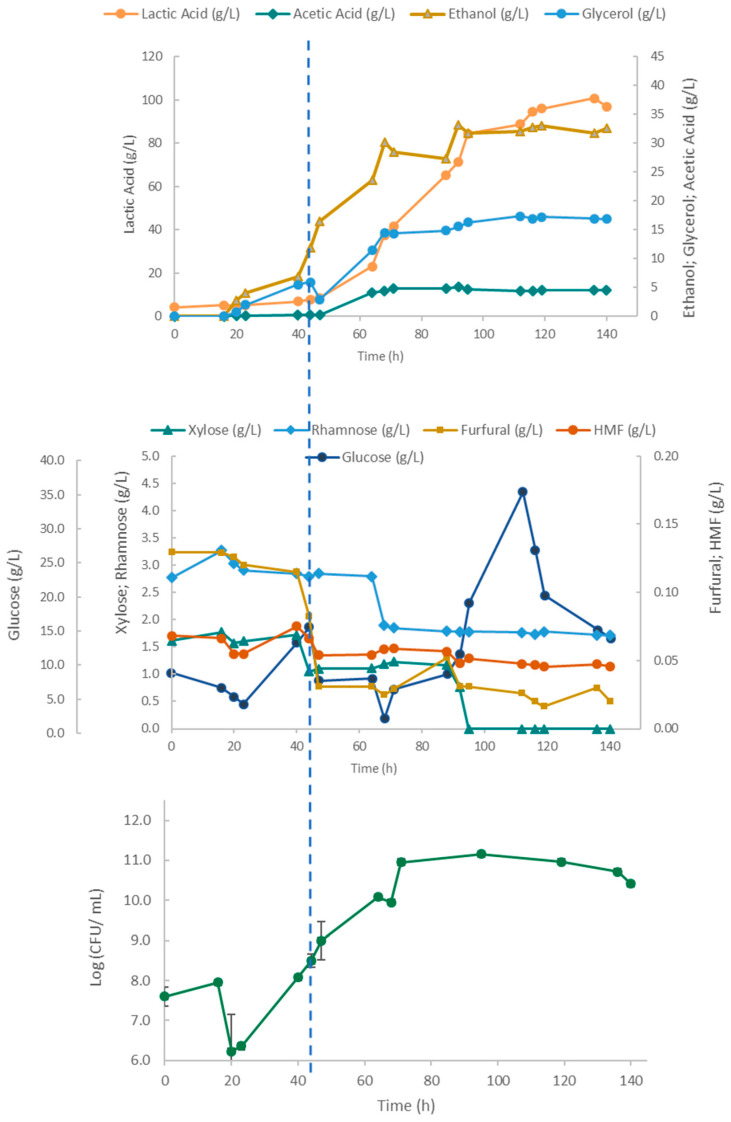
Time course of the 4LAB and *S. cerevisiae* co-cultivation in a 2 L stirred tank bioreactor using *Ulva* hydrolysate in the batch phase (up to 22 h) followed by a fed-batch phase with a concentrated glucose solution as feed (pulses roughly every 20 h). The vertical dashed line indicates the time of 4LAB inoculation.

The results from the fed-batch fermentation of *Ulva* hydrolysate by this co-culture of lactobacilli and yeast are shown in Figure 8.

For the first 16 h of fermentation, the yeast growth was impaired, both by the presence of inhibitors and by the temperature in the bioreactor being set to 37 °C instead of the optimum yeast growth temperature of 30 °C. Also, the stirring speed set initially at 50 rpm may have been inadequate to guarantee a sufficient oxygen supply and mixing of the bioreactor contents. After the corrections were implemented at 15 h, the yeast culture resumed growth, ethanol production, and furan consumption, as shown by the reduction of HMF and furfural levels. At 45 h, the lactobacilli inoculum was introduced, and thereafter, the ethanol and glycerol concentrations increased rapidly, accompanied by a further decrease in the HMF levels. The lactic acid concentrations also increased and reached slightly higher levels (101 g/L) than in the 4LAB monoculture (94.5 g/L, Figure 5). Glycerol, acetic acid, and ethanol levels stabilized at 70 h, suggesting a decline in microbial activity, particularly that of the yeast, with growth arrest. Despite the diminished inhibitory impact of lactic and acetic acids at controlled pH levels, the concentrations they attained can still stress yeast cells, potentially leading to the observed decline of ethanol production. The overall CFU profile initially evidenced the poor environmental conditions for yeast growth described above. From 20 h onwards, the total CFU number gradually increased, peaking at 95 h. Thereafter, the CFU count remained stable, suggesting the arrest of yeast growth at around 70 h of fermentation time, accompanying that of ethanol production.

#### 2.5.3. Yeast Fermentation

**Figure 9:**
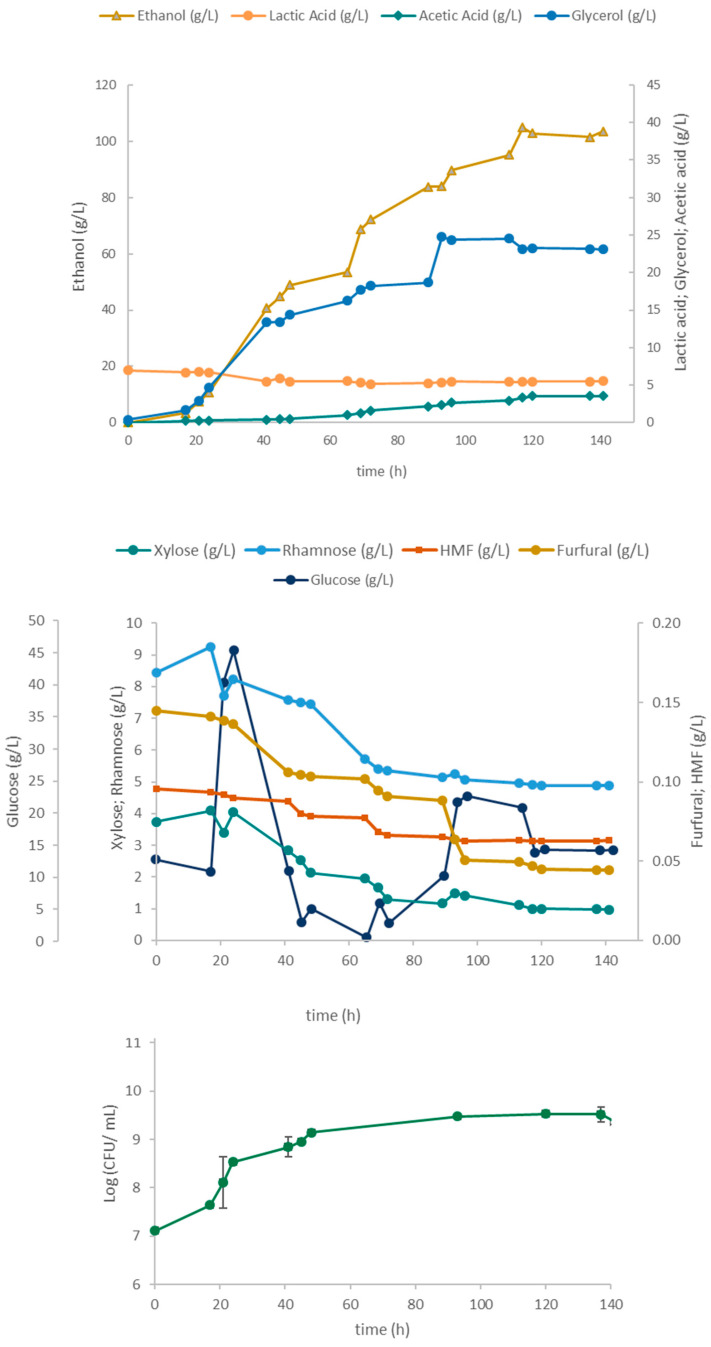
Time course of *S. cerevisiae* cultivation in a 2 L stirred tank bioreactor using *Ulva* hydrolysate in the batch phase (up to 19 h) followed by a fed-batch phase with a concentrated glucose solution as feed (pulses roughly every 20 h).

The time course of *Ulva* hydrolysate fermentation using *S. cerevisiae* as the sole microorganism is presented in Figure 9. Following the lag phase (first 20 h), and upon the onset of glucose feeding, an increase in both CFU counts and yeast metabolite production was observed up to 120 h (Figure 9). The attained ethanol concentrations differed significantly between this yeast culture (105 g/L) and the co-culture in Figure 8 (33.1 g/L). Since CFU counting did not allow for the differentiation between yeast and LAB, a direct comparison between *S. cerevisiae* growth in a monoculture and in a co-culture, in terms of biomass yield, could not be conducted. It is possible that suboptimal conditions for the yeast in a co-culture favor lactobacilli growth over yeast. This is suggested by the similarity in the metabolite production profiles observed in the 4LAB culture (Figure 7) and their counterpart in co-culture with yeast (Figure 8).

In all, despite the presence of inhibitors, the cultivations yielded high cell and metabolite concentrations. Notably, the ability of *S. cerevisiae* to detoxify the *Ulva* hydrolysate was demonstrated by the reduction in time of HMF and, especially, furfural (Figure 8 and Figure 9).

### 2.6. Characterization of Fermented Products

#### 2.6.1. Nutritional Profile Analysis

Fermented products (whole broth) were freeze-dried to preserve their stability and characterized along with the unprocessed form of *Ulva* to examine the effects of the sequential saccharification and fermentation treatments on the nutritional profile.

A comprehensive analysis of the used *Ulva* batches and respective fermented products was undertaken to determine their potential for feed purposes, and the results are presented in Table 2.

#### 2.6.2. Peptide Profile

**Figure 10:**
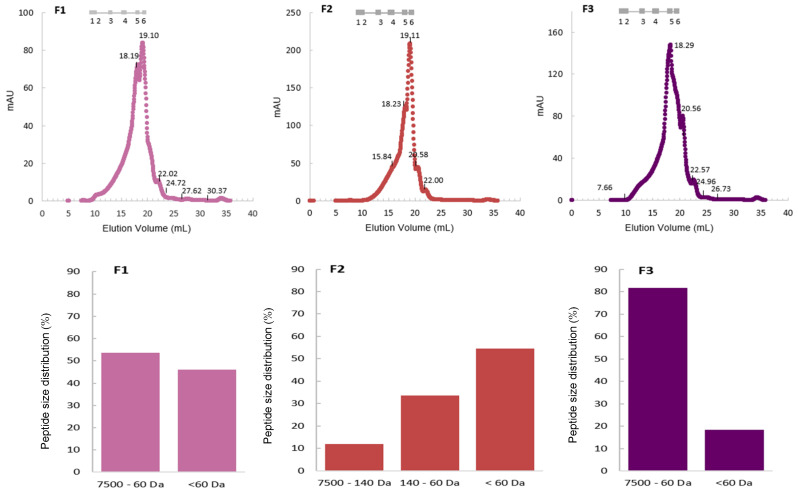
Peptide profile obtained by size exclusion chromatography with column Superdex Peptide 10/300 GL|Cytiva (cytivalifesciences.com) for broths from 4LAB fermentation (F1); 4LAB and yeast co-fermentation (F2); yeast fermentation (F3). Legend top graph: in grey, the elution volumes of the biomolecules used for calibration: 1. cytochrome C (12,384 Da); 2. aprotinin (6512 Da); 3. angiotensin I (1296 Da); 4. (Gly)6 (360 Da); 5. (Gly)3 (189 Da); 6. Gly (75 Da). Legend bottom graph: peptide size distribution (%) calculated from the top chromatogram peak area values.

The molecular weight distribution among the peptides in the fermented products is shown in Figure 10. The top graphs depict a grey scale with the elution volumes of a mixture of standards (molecular weights in the range of 12.4 kDa to 75 Da) along with the size exclusion (SEC) chromatograms obtained with aqueous solutions of the dried fermented broths (F1 to F3). A good fractionation of the soluble sample components was not possible in the selected SEC column, as the molecular size profiles of the peptides are continuous within the resolution capacity of the resin (no isolated peaks). Nonetheless, it is possible to compare fermented broth samples throughout the entire separation range of the column. Based on the chromatographic profiles, a characterization of size distribution was attempted (Figure 10, bottom graphs), for subsequent discussion (Section 3.2.1).

#### 2.6.3. Antioxidant Activity of Fermented Products

The antioxidant activity (DPPH, ABTS radical scavenging, and reducing power), as well as the chelating (“anti-pro-oxidant”) properties for Cu^2+^ and Fe^2+^ ions of the fermented *Ulva* products, were evaluated. From the acquired data (Table 3), it is possible to observe that the fermentation conditions influence the biological activities, particularly in terms of ABTS and DPPH antioxidant activity. In the case of F1 and F2, a QFe EC_50_ value was not attained in the range of concentrations tested.

#### 2.6.4. Protein Bioaccessibility

**Figure 11:**
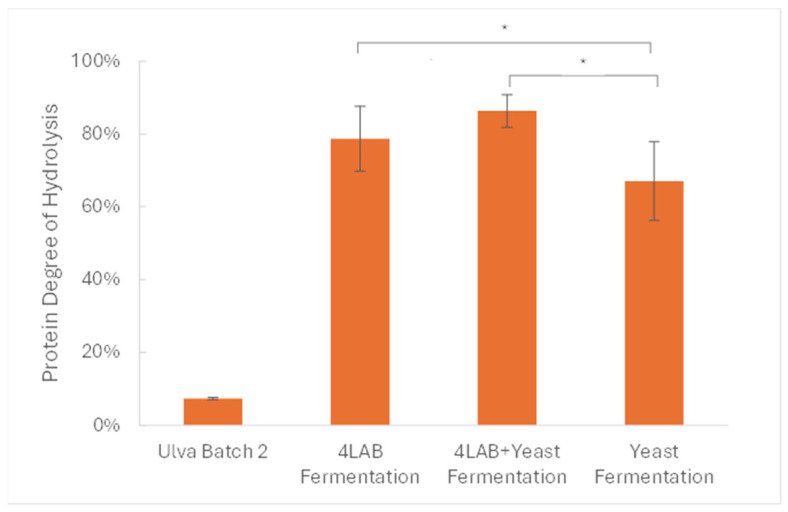
Degree of protein hydrolysis for samples of unprocessed *U. rigida* and its freeze-dried fermentation broths submitted to simulated gastrointestinal digestion. Data presented are the means of triplicates ± standard deviations. Significant differences (* *p* < 0.05) are denoted by significance stars (unpaired two-sample Student’s *t*-test).

To determine protein bioaccessibility, gastrointestinal digestion was simulated, as described in Section 4.9.8. Proteolysis was quantified using the OPA method to measure the primary amine groups exposed after enzymatic hydrolysis, which should increase in concentration. The final degree of hydrolysis for each sample is expressed as a percentage (based on leucine equivalents) of the total amino acid content quantified, as described in Section 4.9.10. As seen in Figure 11, at the end of the simulated digestion, only 7.4 ± 0.3% of the raw *Ulva* protein was hydrolyzed. In contrast, a high degree of hydrolysis can be observed in the fermented broth samples, with values of 78.7 ± 8.9%, 86.3 ± 4.5%, and 67.0 ± 10.8% for the 4LAB broth, mixed consortium (4LAB + yeast) broth, and yeast broth, respectively.

## 3. Discussion

### 3.1. Hydrolysis and Fermentation Results

From the *Ulva* batches analyzed, those with higher carbohydrate content were selected as feedstock for microbial protein production through fermentation. After complete hydrolysis using the NREL protocol, glucose was the sugar present in the highest concentration, followed by rhamnose and xylose. The latter two originated from ulvan, the sulfated polysaccharide typical of *Ulva* species. This hierarchical pattern in terms of sugar concentration was also observed in other studies and is typical of *Ulva* species. As the *Ulva* used in this work was obtained by cultivation, the most significant difference probably lies in the extent of starch hydrolysis. The accumulation of this reserve polysaccharide in the *Ulva* cytoplasm is a result of specific conditions imposed during *Ulva* growth, namely of N limitation (Prabhu et al., 2019) [34].

For seaweed to be used as a source of sugars for microbial growth, its pre-treatment is required for sugar release, as many sugars are not freely available, being instead bound in structural and storage polysaccharides (El Harchi et al., 2018) [35]. For cellulose hydrolysis, a thermochemical treatment to open the cell wall structure, followed by enzymatic hydrolysis, results in the highest glucose concentrations and hydrolysis yields (El Harchi et al., 2018; Maneein et al., 2018) [35,36]. For the complete hydrolysis of ulvan to its monomers, rhamnose, xylose, and glucuronic and iduronic acids, a specific enzymatic cocktail including ulvan lyases is needed. As this enzymatic cocktail is not commercially available, ulvan hydrolysis was partially attained in the present work by using a thermochemical step with sulfuric or hydrochloric acids at different concentrations. Figure 3 and Table 4 show that, at similar concentrations, hydrolysis with HCl increased the total sugars, except at the 5% (*w*/*v*) level, which leads to sugar degradation to furan compounds. Furans like HMF and furfural are known to inhibit microbial growth, and their combined presence generally amplifies this effect. Thus, furan concentrations near the inhibitory levels must be avoided. Concentrations of 0.5 to 1 g/L for HMF and furfural were found to be toxic to lactic acid bacteria (Zhang et al., 2016) [37]. Even though HCl treatments at lower concentrations (3% *w*/*v*) yielded higher values of total released sugars (TRS = 23.1 g/L) and TRS yield (54%) in comparison to H_2_SO_4_, they resulted in higher productions of inhibitory compounds. For this reason, HCl pre-treatments were rejected, and H_2_SO_4_ was adopted. After sulfuric acid treatments at 1% and 3% (*w*/*v*), an enzymatic treatment using a cellulase cocktail was carried out to check if glucose release could be further enhanced. In both situations, an increase in the glucose concentration was observed, with the effect being greater after treatment with 1% H_2_SO_4_ (>2-fold increase) than with 3% H_2_SO_4_ (>1.4-fold increase) because the cellulose had already been partly hydrolyzed during the 3% H_2_SO_4_ treatment.

Shake flask cultivation assays using clarified *Ulva* hydrolysates prepared by either acid pretreatment with 1% H_2_SO_4_ followed by cellulase hydrolysis, or just acid pre-treatment with 3% H_2_SO_4_ or 5% H_2_SO_4_, were carried out to test their potential as carbon sources for the growth of the 4LAB consortium or *Saccharomyces cerevisiae*.

In the fermentation with the 4LAB consortium, not only lactic acid but also acetic acid and ethanol were produced, demonstrating the heterofermentative nature of some of the LAB species. Glucose was the first sugar to be consumed, but rhamnose and xylose were also uptaken from the hydrolysates prepared with 3 and 5% H_2_SO_4._ In all cases, xylose and rhamnose were most probably consumed by *L. brevis* and *L. rhamnosus* (Hwang et al., 2011) [38]. Moreover, this uptake happened at the beginning of the assays when glucose was still present at high concentrations. In LAB, the utilization of different carbohydrates is usually governed by the carbon catabolic repression (CCR) system, namely in *L. plantarum*, *L. casei*, *L. delbrueckii*, and *L. pentosus* (Andreevskaya et al., 2016; Kim et al., 2009) [39,40]. It is worthy of note that simultaneous carbohydrate utilization has been demonstrated in a few species, including *L. brevis*, which exhibits simultaneous utilization of xylose and glucose through the heterofermentative pathway.

Shake flask cultivation assays were also carried out with *Saccharomyces cerevisiae* (Figure 6). In addition to ethanol, glycerol was produced and attained higher values in the case of the harsher chemical pre-treatment with 5% (*w*/*v*) H_2_SO_4_. Glycerol is both a byproduct of ethanol production and a major osmolyte generated during hyperosmotic stress. Its synthesis and accumulation allow yeast cells to avoid dehydration by balancing intracellular osmolarity with that of the environment (Aslankoohi et al., 2015) [41].

Similar to lactobacilli, *S. cerevisiae* is able to partially metabolize furfural and HMF. *S. cerevisiae* fermentation has been shown to be impaired by concentration ranges of 1–5 g/L of HMF (Taherzadeh et al., 2000) [42] and 0.5–4 g/L of furfural (Banerjee et al., 1981) [43]. Nonetheless, this yeast has demonstrated the capability to convert HMF and furfural into less-inhibitory compounds if their concentrations are below lethal levels [42]. Under anaerobic conditions, HMF and furfural are mainly converted to their corresponding alcohols, 2,5-furandimethanol and furfuryl alcohol, respectively, while in aerobic conditions furfural is converted to furoic acid (Ask et al., 2013) [44]. These compounds were not detected in the present study, probably due to the low initial concentrations of HMF and furfural. However, the detoxifying capabilities of both *S. cerevisae* and lactobacilli were well demonstrated by the experimental data, as the concentrations of both furans in the cultivation assays with 3% and 5% H_2_SO_4_ hydrolysates decreased throughout the culture time (Figure 5 and Figure 6).

The total concentration of sugars released during the 3% (*w*/*v*) H_2_SO_4_ treatment was lower in the hydrolysates used for the mixed cultivation (4LAB + yeast). This might have occurred because a different batch of *Ulva* was used in these assays. Thus, to assess the adequacy of the three different hydrolysates as substrates for LAB and yeast growth, the yield of lactic acid and ethanol on the total consumed sugars was considered a more useful parameter for comparison and is given in Table 5. From the results, the yields of lactic acid and ethanol on the total sugars tend to decrease the harsher the treatment is and are lowest for the hydrolysates prepared with 5% H_2_SO_4_, probably because of a higher concentration of inhibitors. An exception is the yield of lactic acid on the total consumed sugars obtained with the hydrolysate prepared with 3% (*w*/*v*) H_2_SO_4_. Although the initial total amount of sugars was lower, the attained concentration of lactic acid was comparable to that from the hydrolysate prepared with 1% H_2_SO_4_ + enzymatic hydrolysis, 13.4 g/L versus 14.8 g/L, respectively. A possible reason might have been the consumption of the other components present in the CSL, e.g., amino acids, upon the exhaustion of glucose, and thus, these results must be considered with caution.

The preliminary assays in the shake flasks demonstrated the potential of the algal hydrolysate as a substrate for single-cell protein production. Subsequent assays were designed to use the whole algal slurry (sugar-rich solution and remaining solids), after hydrolysis, as a substrate for fermentation with 4LAB and/or yeast to produce a seaweed-based fermented product that is a mixture of microbial biomass and hydrolyzed *Ulva*. This product is expected to provide higher nutrient bioaccessibility after the fiber hydrolysis step, an enriched nutritional profile derived from microbial fermentation (different proteins, vitamins, essential amino acids, and lipids), and improved bioactivities.

The scale-up of *Ulva* hydrolysate fermentations took place in a 2 L stirred tank bioreactor. Acid hydrolysis with 3% (*w*/*v*) H_2_SO_4_ was chosen as the pre-treatment. Although the yield of hydrolysis was lower when compared to the combined treatment with cellulases (Figure 4 and Table 4) and lower than that of acid hydrolysis with 5% (*w*/*v*) H_2_SO_4_, it was considered a good compromise, as it avoided both the use of enzymes (at extra cost) and a too harsh acid treatment which released higher concentrations of inhibitors.

Three different fermentations, namely using the 4LAB consortium, *S. cerevisae*, and a co-culture of both, were carried out. The whole *Ulva* slurry from acid hydrolysis was used as a source of sugars and peptides/amino acids for microbial growth. The whole fermented broths (final slurry with both algal and microbial biomasses) were freeze-dried for subsequent characterization. In the cultivation with the 4LAB consortium, the lactic acid and acetic acid levels increased significantly after 23 h of fermentation. The lactic acid titer rose gradually up to 94 h, attaining a value of 95 g/L, while the acetic acid and ethanol increased until the end of the process, attaining values of approximately 4 g/L. Lactic acid ceased being produced when cell growth was arrested. This result might be associated with the inhibition induced by the high osmotic pressure caused by the accumulation of lactate in the medium, as has been reported (Cui et al., 2016) [45]. In addition to being a food preservative, lactic acid (LA) also has biological properties such as antimicrobial and immunomodulatory ones. The use of an alternative to the standard MRS medium did not have a negative impact, as high metabolite titers and glucose consumption were achieved.

The assays using a co-cultivation of *S. cerevisiae* and the 4LAB consortium yielded a slightly higher lactic acid concentration of 101 g/L and an ethanol concentration of 33 g/L. Some studies have demonstrated the positive effect of co-cultivating yeast and LAB on lactic acid synthesis. During an investigation into cocoa fermentation, it was found that alcoholic conditions heightened the yields of lactic and acetic acids from LAB, raising the possibility of an interaction between yeasts and LAB, which are crucial for acid synthesis (Ouattara et al., 2019) [46]. In the present study, ethanol and LA were produced simultaneously, although ethanol production was arrested much earlier (at circa 70 h) when lactic acid concentration was approximately 40 g/L. However, the total CFU count gradually increased, peaking at 95 h. As the total cell count included both LAB and yeast, the active growth of lactobacilli may have outweighed the decline of viable yeast cells. To better understand the growth dynamics during co-fermentation, specific plates for *S. cerevisiae* growth, such as YPD agar plates, should have been used alongside the MRS plates for LAB growth.

Concerning the fermentation with only yeast, ethanol and glycerol were produced up to circa 92 h of cultivation, when the CFU count also stabilized, indicating no benefits in extending fermentation for the purpose of microbial growth. The final CFU count was lower than in the 4LAB fermentation, despite the similar end-product concentrations (95 g/L of lactic acid, Figure 7; 105 g/L of ethanol, Figure 9). This outcome may be explained by the initial inoculum size and the lower specific growth rates of yeast when compared to LAB. Ethanol ceased being produced at circa 120 h when it attained an inhibitory concentration of 105 g/L. According to the supplier, ethanol concentrations above 90–110 g/L are inhibitory to this strain.

In all, despite the presence of inhibitors, the experiments yielded high microbial biomass and metabolite concentrations. The ability of *S. cerevisiae* to detoxify the hydrolysate was particularly remarkable. This can be observed in Figure 8 and, as already mentioned, was reported before [42,44].

### 3.2. Characterization of Raw Ulva and the Fermented Products

#### 3.2.1. Proximate Composition and Peptide Profile

Table 2 gives the proximate composition of two batches of cultivated *Ulva rigida* biomass and of their freeze-dried products after fermentation. The composition of *Ulva* biomass is similar to that found in the literature [47,48] for carbohydrates, ashes, and protein, except for the protein content in batch 2 (5.4 ± 0.5% dw), which was particularly low. Concerning the fermented products, it can be observed that *Ulva* glucans, after acid hydrolysis, were extensively consumed in all fermentations and that LAB efficiently utilized xylose and rhamnose for growth and metabolite synthesis, since there were no measurable levels of these compounds in the respective fermented broths. Yeast-fermented products, on the other hand, still contained unconsumed rhamnose and xylose. LAB-fermented products presented an LA concentration of approximately 4% dw. Although high lactic acid contents may raise concerns in aquafeeds, it is important to consider that only a small percentage of the herein-produced ingredients would be incorporated as protein ingredients in aquaculture feeds. Ethanol, on the other hand, despite the high concentrations in the final broth of yeast fermentation (circa 100 g/L), was not detected in the processed samples due to its high volatility and subsequent evaporation during the lyophilization process. Each of the three types of fermentation that were tested was able to increase the protein content when compared to the unprocessed *Ulva*, namely 1.1-fold with the fermentation by LAB, 1.3-fold using the mix 4LAB + *S. cerevisiae*, and 4.4-fold using the *S. cerevisiae* fermentation (*Ulva* batch 2 was used in this fermentation).

The implementation of different fermentation conditions is anticipated to yield different effects on the composition of proteins and peptides. Bioactive peptides are initially inert within their precursor molecules but can be activated after release through processes such as in vivo gastrointestinal digestion, in vitro hydrolysis, or microbial fermentation (Q. Guo et al., 2023; Jakubczyk et al., 2020) [49,50]. These peptides generally consist of 2 to 20 amino acid residues that exhibit diverse biological properties depending on their specific amino acid composition, sequence, and structure (Du and Li, 2022) [51]. Considering that the average amino acid molecular weight is circa 100 Da, then, the peptides within the size range of 200 Da to 2000 Da might exhibit beneficial biological effects (Karami et al., 2019) [52]. Moreover, LABs hold considerable potential to produce a substantial quantity of bioactive peptides due to their proteolytic system (Q. Guo et al., 2023) [49].

The products derived from both 4LAB fermentations (mono- and co-culture) exhibit very similar molecular size profiles, although the combined fermentation allowed for a better differentiation of peptide sizes. The major difference between the samples was the absence of a peak at the 19 mL elution volume in the yeast-fermented product, which corresponds to di- and/or tripeptides in terms of molecular weight. The yeast broth showed the highest percentage of 60 to 7500 Da MW peptides, at approximately 82%, followed by the co-culture with 58%, and finally the 4LAB broth with 54%. However, due to the broad range of this interval, it is not possible to conclude through this analysis which product is potentially richer in bioactive peptides. Additionally, all samples presented molecules with a molecular weight below 60 Da. These molecules do not correspond to amino acids, as the smallest amino acid, glycine, has a molecular weight of 75 Da. Hence, it is plausible that substances other than peptides, proteins, and amino acids were also detected at the wavelength λ = 280 nm used for SEC peak quantification.

#### 3.2.2. Antioxidant Activity of Fermented Products

From the results, the fermented products from the co-culture and yeast fermentations show slightly higher antioxidant activities in terms of ABTS and reducing power but performed less in terms of DPPH scavenging when compared to that of the 4LAB culture. The combination of LAB and yeast seems to generate compounds capable of bioactivity synergism. However, yeast-fermented seaweed still showed stronger chelating properties and reducing power when compared to the co-culture fermentation product. Additionally, only the product from yeast fermentation exhibited significant chelating activity towards iron ions in the range of concentrations tested. Antioxidant activities, in terms of ABTS and reducing power and Cu^2+^ and Fe^2+^ chelating power, were also evaluated for *Ulva* aqueous extracts. The attained ABTS EC_50_ values and reducing power were 19.45 mg/mL and 17.75 mg/mL, respectively, while the Cu^2+^ chelating power was 9.30 mg/mL. Compared to the aqueous extracts of *Ulva*, the fermented products present a notably higher antioxidant activity.

Enhanced antioxidant activities may be attributed to the hydrolysis or breakdown of algae cell walls by the acid treatment, resulting in the release of various antioxidant compounds such as phenolics and flavonoids. Furthermore, the production of microbial secondary metabolites can contribute to beneficial biological effects, since several biochemical reactions that take place during fermentation, such as decarboxylation, hydrolysis, and esterification processes, potentially generate active ingredients (Hur et al., 2014) [53]. Fermentation also induces the structural degradation of proteins, resulting in the release or synthesis of various compounds that exhibit iron-chelating activity. It is crucial to analyze the nature of the compounds that exhibit natural antioxidant and chelating properties, particularly when considering the application of these products as a feed supplement. For example, as previously mentioned, phenolic compounds are known to possess antioxidant effects. However, these chemicals tend to form complexes with proteins and inhibit digestive enzymes, impairing the functional and nutritional properties of proteins. Consequently, these compounds are generally undesirable in food and feed products (Sim et al., 2021) [54]. For this reason, it is important to determine the bioaccessibility of the protein in the final products.

#### 3.2.3. Protein Bioaccessibility

The high degree of protein hydrolysis shown in all of the fermented samples when compared to the raw *Ulva* biomass indicates a high protein bioaccessibility. Furthermore, broths containing LAB strains presented a higher degree of hydrolysis, suggesting that fermented products with these strains had higher protein bioaccessibility than yeast-fermented products. This indicates that the proteins in the LAB samples were more prone to being hydrolyzed by the gastrointestinal (GI) enzymes and consequentially to being absorbed in the GI tract. These results should, however, be confirmed in in vivo studies, due to differences in gastrointestinal conditions in different target species.

## 4. Materials and Methods

### 4.1. Raw Materials

Several *Ulva rigida* batches were purchased from the algae producer ALGAplus Lda (Ílhavo, Portugal). ALGAplus produces *Ulva rigida* in an open land-based integrated multi-trophic aquaculture (IMTA) system located at the Ria de Aveiro coastal lagoon. The biomass was received already washed with salt water, dried, and milled into flakes measuring circa 1.5 mm. It was stored and protected from light and humidity.

### 4.2. Microorganisms

A consortium of four lactic acid bacteria strains, namely *L. casei* ATCC393, *L. rhamnosus* ATCC 7469, *L. brevis* DSM 20054, and *L. plantarum* ATCC 8014, were selected in virtue of their capability to metabolize the different monosaccharides released after the hydrolysis of different types of seaweed. As for yeast, the *S. cerevisiae* strain SafAle^TM^ US-05 (Lesaffre, Lille, France) was chosen on the basis of its beneficial contribution to fermented foods. Moreover, these microorganisms were selected for the organoleptic and preservation properties provided to their fermented products and, finally, for being GRASs.

All microbial strains were stored at −80 °C. Stock cultures of each *Lactobacillus* species were prepared under aseptic conditions by transferring 1.5 mL of inoculum in the exponential growth phase grown in De Man, Rogosa, and Sharpe (MRS) broth (PanReac Applichem) to 2 mL sterile cryovials with 300 μL of previously sterilized glycerol. A similar procedure was carried out to prepare *S. cerevisiae* stocks, except that this yeast was previously grown in yeast extract–peptone–dextrose (YPD) broth.

### 4.3. Chemical Pre-Treatment

*Ulva rigida* biomass chemical pre-treatment consisted of acid hydrolysis for 30 min and at 121 °C while varying the acid type and concentration. Dilute acid treatments with H_2_SO_4_ and HCl at 0.5%, 1%, 3%, and 5% (*w*/*v*) were implemented for a 10% (*w*/*v*) seaweed biomass load. After hydrolysis, the pH of the *Ulva* slurries was adjusted to 4.8 using an 8 M NaOH solution, and the solubilized sugar monomers and inhibitory microbial compounds were quantified by high-performance liquid chromatography (HPLC).

### 4.4. Enzymatic Treatment

For cellulose hydrolysis, enzyme preparations from Novozymes^®^ (Novozymes, Bagsvaerd, Denmark) Celluclast BG with an activity of 2840 FPU/g (Hegedús et al., 2012) [55] and β-glucosidase (NS 22118) with an activity of 297.2 U/mL were combined. Celluclast BG activity was determined according to Ghose 1987 [56], while that of β-glucosidase (NS 22118) was determined using the p-nitrophenyl-β-D-glucopyranoside-pNPG protocol. In this protocol, one unit (U) of enzyme activity is defined as the amount of β-glucosidase that produces 1 μmol of pNP per minute (Hang and Woodams 1994) [57]. The amounts of enzymes added were 1.3 mg/mL_biomass suspension_ for Celluclast and 0.75 µL/mL_biomass suspension_ for β-glucosidase, corresponding to 3.7 FPU/mL_biomass suspension_ and 0.22 U/mL_biomass suspension_, respectively.

After diluted acid hydrolysis and under aseptic conditions, the pH of the algal slurry was adjusted to 4.8 using an 8 M NaOH solution. Enzymatic treatment was conducted in an orbital incubator, with a stirring speed of 200 rpm at 50 °C for 30 h. A sample was taken from each flask, before and after enzyme addition, so that the contribution of the chemical pre-treatment to sugar release could be considered. Then, throughout the 30 h, samples were collected every 2 h and analyzed by HPLC.

### 4.5. Seed Medium and Inoculum Preparation

Two different inocula were prepared: a lactic acid bacteria consortium (4LAB) encompassing *L. rhamnosus*, *L. brevis*, *L. casei*, and *L. plantarum* strains and an axenic *Saccharomyces cerevisiae.* The four LAB strains were pre-cultivated separately in an MRS medium under orbital agitation at 37 °C and 100 rpm. *S. cerevisiae* was cultivated in YPD medium at 30 °C and 250 rpm. Flasks containing 80 mL of culture medium for LAB and 20 mL for *S. cerevisiae* were inoculated directly with 1.8 mL cryovials taken from the cell bank. Cells were harvested after 16 to 18 h of growth, corresponding to the end of the exponential growth phase. For the shake flask growth assays on the *Ulva* hydrolysates, each inoculum was prepared by transferring the necessary volume of pre-inoculum so that the shake flask cultures were started with an optical density (OD_600nm_) of 0.5. In the bioreactor experiments, the volume pertaining to each LAB strain was calculated to achieve an OD_600nm_ of 0.2, at the beginning of the bioreactor fermentation assay, and for the yeast, an OD_600nm_ of 0.7 was chosen. For both assays, the required culture volume was collected and centrifuged at 6000× *g* and 4 °C for 15 min. Afterward, the supernatants were discarded aseptically, and the pellets were resuspended in a sterile 0.5% (*w*/*v*) NaCl solution to serve as the inoculum to the growth media.

### 4.6. Shake Flask Fermentations

Three types of *Ulva* hydrolysates were used in the fermentation assays, namely the hydrolysate prepared after chemical pre-treatment with 1% sulfuric acid followed by enzymatic treatment and the single chemical pre-treatments with 3% and 5% sulfuric acid. The hydrolysates were centrifuged (6000× *g*, 10 min) and vacuum-filtered using a Buchner funnel equipped with a 900 μm pore-sized membrane. Mineral components and corn steep liquor (CSL) were added to the obtained filtrates, resulting in the following formulation: 830 mL/L *Ulva* filtrate, 40 mL/L CSL, 2 g/L di-ammonium hydrogen citrate, 0.2 g/L MgSO_4_, and 0.05 g/L MnSO_4_. The pH of the culture medium was adjusted to 6.2–6.5 using solutions of 1 M HCl and 8 M NaOH before inoculation. The growth assays with the 4LAB consortium and *S. cerevisiae* in the different test conditions were conducted in duplicate for 50 h, with orbital shaking at 37 °C and 100 rpm and 30 °C and 250 rpm, respectively.

### 4.7. Bench-Scale Bioreactor Fermentations

The scale-up of fermentation on the *Ulva rigida* hydrolysates was conducted in a 2 L bioreactor, and data acquisition was carried out with the software BioCommand/SCADA (BioFlo115, Eppendorf AG, Hamburg, Germany). The bioreactor was assembled, filled with 900 mL of distilled water, and autoclaved at 121 °C for 25 min. A new batch of *Ulva* pre-treated with 3% H_2_SO_4_ was prepared separately and used in each fermentation run as the main component of the base culture medium. This *Ulva* slurry was added to the sterilized and emptied bioreactor along with all the previously sterilized remaining components of the cultivation medium. All assays started in batch mode with a 1.3 L working volume of the base cultivation medium and, before glucose exhaustion, were changed to fed-batch mode using a 500 g/L sterile glucose solution as the feed solution.

#### 4.7.1. Fermentation with 4LAB

Fermentation of *Ulva* biomass with the 4LAB consortium was carried out for 140 h, with the dissolved oxygen (DO) set at 5% of saturation and a pH set point of 6.5 maintained with the controlled addition of a 30% (*w*/*v*) NH_4_OH solution. A cascade control of DO was set with lower and upper limits of agitation speed, at 50 and 600 rpm, respectively, and a target air supply flow rate of 0.65 L/min that was lowered to 0.3 L/min at 46.4 h due to consistently high DO readings.

#### 4.7.2. Co-Fermentation with 4LAB + Yeast

A mixed fermentation of *Ulva* hydrolysate was conducted by first inoculating the medium with *S. cerevisiae*. The initial working parameters were set to 37 °C, 50 rpm agitation speed, and pH 6.5. At 14.8 h of fermentation, temperature and stirring speed were changed to 30 °C and 200 rpm, respectively, as the yeast was barely consuming the carbon source. Once yeast growth took up (circa 23 h), the initial bioreactor conditions were restored, and at 46.5 h of fermentation, the 4LAB consortium was inoculated into the culture medium. The dissolved oxygen was maintained at 5% saturation by using a cascade control on the agitation speed with lower and upper limits of 50 and 600 rpm, respectively.

#### 4.7.3. Fermentation with Yeast

Fermentation of *Ulva* biomass with *S. cerevisiae* was carried out for 141 h using 0.65 L/min of air supply, temperature of 30 °C, and pH 5.5. The set point for DO was 5% saturation. A cascade control of DO was set with the stirring speed at lower and upper limits of 50 and 600 rpm, respectively.

### 4.8. Processing and Analysis of Fermented Ulva

The fermented *Ulva* slurries were lyophilized for 72 h. The recovered material was manually ground into powder and kept at room temperature in a desiccator until quality analyses were carried out.

### 4.9. Analytical Methods

#### 4.9.1. Total Carbohydrates in Seaweed Biomass

Total carbohydrates in the *U. rigida* flakes were quantified in triplicate by the NREL protocol “Determination of Total Carbohydrates in Algal Biomass” (Wychen et al., 2013) [58], with an adjustment of the sample weight as described in Mateus et al., 2024 [59].

#### 4.9.2. Quantification of Sugars and Organic Acids

Offline determination of the sugars and fermentation metabolites in the liquid samples was carried out in a HPLC system (Hitachi LaChrom Elite, Hitachi High-Tech Science Corporation, Tokyo, Japan) equipped with a Hitachi L-2490 refraction index (RI) and a Hitachi L-2420 UV-Vis VIS detector. A Rezex ROA-Organic acid H+ 8% column was used and kept at 65 °C. A mobile phase of 5 mM H_2_SO_4_ at the flow rate of 0.5 mL/min and an injection volume of 20 μL was used. Samples were treated and analysis occurred as described in Mateus et al., 2024 [59].

#### 4.9.3. Viable Cell Counting

The colony-forming unit (CFU) method was used for viable cell count determination throughout bioreactor cultivations. Thereupon, 100 μL of homogenized samples were diluted with 900 μL of sterile NaCl 0.85% (*m*/*v*), and successive 1:10 dilutions were prepared under aseptic conditions. For three selected dilutes, 50 μL of the sample were transferred to MRS–agar Petri dishes, in triplicate. For cell counting in LAB-containing cultures, agar plates were incubated for 48 h at 37 °C and kept at 4 °C until counting. In axenic yeast cultures, plates were incubated for 48 h at 30 °C. Although not being the most adequate medium for yeast growth, a previous test showed that the yeast cells also grew on MRS agar plates.

#### 4.9.4. Protein Content

For the determination of total protein content in the lyophilized samples, a 96-well plate assay adaptation of the Lowry method was used, as described by the Thermo Scientific protocol [60] with some modifications. In a 1 mL microtube, a 20 mg/mL solution of the sample was prepared in 2 N NaOH and hydrolyzed in an oven at 100 °C for 20 min with occasional stirring and finally centrifuged for 5 min at 9600× *g* at room temperature. Standard samples were prepared using BSA in 6 different concentrations in the range 125–1500 µg/mL. To each well of a 96-well plate (Greiner, Ref:655101), 40 µL of each sample and a further 200 µL of modified Lowry reagent were added before incubation in the microplate reader (Thermo Scientific™ Multiskan™ GO, ThermoFisher Scientific, Waltham, MA, USA) for 10 min at room temperature. After the incubation period, 20 µL of 1N Folin–Ciocalteu’s Reagent (Sigma Aldrich, Sigma Aldrich Corporation, St. Louis, MO, USA) were added to each well. The plate was then mixed for 1 min in the microplate reader and incubated for 30 min. The absorbance of the samples was measured at 550 nm and 750 nm. Blanks for each sample were prepared by substituting the Folin–Ciocalteu’s Reagent with water for the determination of interferences at the measured wavelengths.

#### 4.9.5. Total Solids and Ash Contents

Total solids and ash contents were determined using an adapted protocol based on the analytical procedure “Determination of Total Solids and Ash in Algal Biomass” by Wychen et al., 2013 [61]. The followed protocol was described recently by Mateus et al., 2024 [59].

#### 4.9.6. Peptide Profile

The peptide profile of the fermented and freeze-dried products was examined using size exclusion chromatography with a fast protein liquid chromatography system (FPLC) AKTA (Amersham Biosciences, Uppsala, Sweden). Peptide detection was performed using a Monitor UPC-900 (AKTA) (Amersham Biosciences, Uppsala, Sweden) set at a wavelength of 280 nm.

Samples from the fermented products F1, F2, and F3 were suspended in distilled water to a concentration of 10 mg/mL and filtered with a 0.22 µm filter to remove the insoluble matter. For the analysis, a gel filtration column Superdex Peptide 10/300 GL from Cytiva ™ (Cytiva, MA, Massachusetts, USA) 10 mm × 310 mm; 24 mL bed volume was used. This column was operated at room temperature, with a flow rate of 0.45 mL/min, using a 30% acetonitrile solution with 0.1% trifluoroacetic acid (*v*/*v*) as an eluent and an injection volume of 500 μL. It is suitable for the detection of peptides and other small biomolecules with molecular weight values between 100 and 7000 Da.

A calibration curve was established, relating molecules’ Kav values (Equation (1)) with the decimal logarithm of their molecular weights, log (MW). Known proteins, peptides, and amino acids, namely, cytochrome C (12,384 Da), aprotinin (6512 Da), angiotensin I (1296 Da), (Gly) 6 (360 Da), (Gly)3 (189 Da), and Gly (75 Da), were used to construct this calibration. The parameter Kav represents the ratio between the elution volume of each molecule and the total volume of the column:

Kav = Ve − V_0_/Vi = Ve − V_0_/(Vt − V_0_) (1)

where Ve is the elution volume of the molecule; V_0_ is the void volume of the column; Vi is the volume within the beads; and Vt is the total bed volume of the column.

A characterization of the size distribution of the peptides in the samples (Figure 10, bottom graphs) was carried out as described in Mateus et al., 2024 [59].

#### 4.9.7. Antioxidant Activity of Fermented Products

Assessments of the antioxidant activity and metal ion chelating power of unprocessed *Ulva* and freeze-dried fermented *Ulva* hydrolysates were conducted. The antioxidant activity was determined through EC_50_ for 2,2′-azino-bis(3-ethylbenzothiazoline-6-sulfonic acid) (ABTS) and 2,2-diphenyl-1-(2,4,6-trinitrophenyl) hydrazyl (DPPH) radicals. The same principle was applied to evaluate the reducing power and metal chelating activity of the samples, with copper and iron ions’ chelation serving as indicators of metal chelation (Henriques et al., 2021) [62]. The procedure described in Mateus et al., 2024, [59] was followed.

#### 4.9.8. Protein Bioaccessibility

To determine protein bioaccessibility, in vitro gastrointestinal digestion was simulated according to Brodkorb et al. [63], with slight modifications. Simulated gastric fluid (SGF) and simulated intestinal fluid (SIF) were prepared accordingly. For the gastric phase, four parts SGF and one part containing Pepsin (Ludovino and Filha, Lda., Lisboa, Portugal; 2000 U/mL), CaCl_2_ (0.075 mM), and HCl were added to a solution of 2.5 mL containing water and enough lyophilized sample to reach 16 mg/mL of protein concentration. A final volume of 5 mL and pH 3 was achieved in each reaction vessel before it was set to incubate at 37 °C for 2 h at 200 rpm in an orbital incubator (Argitorb, Aralab, Rio de Mouro, Portugal). For the intestinal phase, four parts of SIF and one part of Pancreatin (Sigma Aldrich; Trypsin 100 U/mL), Bile salts (Sigma Aldrich) (10 mM), and NaOH were used. A final volume of 10 mL and pH 7 was achieved in each reaction vessel before it was set to incubate at 37 °C for another 2 h at 200 rpm. After digestion, 1.9 mL aliquots were collected, and 100 µL of 20% (*w*/*v*) SDS was added to each one. These were placed in a dry bath at 80 °C for 10 min and thereafter centrifuged for 5 min at 9600× *g*. All the obtained supernatants were analyzed in triplicate through the OPA method (Section 4.9.9).

#### 4.9.9. Primary Amine Determination and Degree of Hydrolysis

The degree of protein hydrolysis was determined through the quantification of primary amines by the OPA method [64]. The OPA reagent was prepared according to the method described by Nielsen et al. [65]. Leucine was used as a standard with concentrations in the range of 0.4–3.2 mM. The spectrophotometric assay was performed in a 96-well plate (Greiner, Ref: 655801), where 180 µL of freshly prepared OPA reagent were added to each well, followed by the addition of 20 µL of sample, blank or standard. The microplate was incubated in the microplate reader (Thermo Scientific™ Multiskan™ GO) with agitation at room temperature for 2 min, after which the absorbance was measured at a wavelength of 340 nm.

#### 4.9.10. Total Protein Hydrolysis

Total protein hydrolysis was performed according to Rutherfurd and Gilani [66]. In a test tube, 20 mg of each lyophilized fermented product were placed, and 1 mL of 6 M HCl was added. Then, nitrogen gas was flushed into the tubes to create an inert atmosphere, and the tubes were tightly closed before being placed in an oven at 110 °C for 24 h. Afterward, the content of each tube was neutralized with 6 M NaOH, and the tubes were centrifuged for 5 min at 9600× *g*. The supernatant was collected for determination in triplicate of the primary amine content, as described in Section 4.9.9.

## 5. Conclusions

Acid treatment with 3% (*w*/*v*) H_2_SO_4_ was selected as the most adequate saccharification method to manufacture sugar-rich *Ulva* hydrolysates, as it resulted in a reasonable yield of polysaccharide hydrolysis with low inhibitor titers. A combined hydrolysis with a subsequent enzymatic step with a cellulase cocktail, although improving the yield of cellulose hydrolysis and increasing TRS, was not chosen due to the additional cost. The selection of this acid treatment was possible due to the proven tolerance of lactobacilli and yeast to the levels of the inhibitors formed, namely HMF and furfural.

Submerged fed-batch bioreactor cultivations using *Ulva* hydrolysates in the initial batch phase and, subsequently, a feed of concentrated glucose solution resulted in fermented products with a higher protein content, a more bioaccessible protein fraction, and antioxidant activity when compared to the original algal biomass. If *Ulva* hydrolysates had been used as a feed in the fed-batch phase, a build-up of inhibitors in the media could have occurred, possibly yielding lower levels of microbial biomass and the respective metabolites. Assays using *Ulva* hydrolysates as feed in fed-batch fermentations are, thus, planned, since if successful, this latter fermentation pattern will much benefit process economics.

*Ulva* fermented products showed increased protein bioaccessibility when compared to unprocessed *Ulva* seaweed. Furthermore, the protein bioaccessibility of the LAB-fermented product is higher than that of the yeast-fermented products, indicating that proteins in LAB broths are more prone to being absorbed in the GI tract. These results should however be confirmed by in vivo studies.

All fermented products showed a higher protein content when compared to the unprocessed seaweed, particularly the yeast-fermented product, even though the protein content was similar amongst the fermented products. For process improvement, other GRAS microbial strains that lead to a higher biomass yield and productivity should be selected. The work with other protein-richer strains is currently being addressed. On the other hand, the recovery of valuable metabolites, e.g., ethanol and lactic acid, from the fermented slurries could potentially favor process economics and further improve the quality of the resulting fermented products in view of their use in feed formulations.

The studies in this work are exploratory in nature but clearly contribute to developing transformed algal products to replace fishmeal in aquaculture formulations. Further, the evaluation of the actual potential of the products should necessarily be assessed in real fish-feeding trials.

## Figures and Tables

**Table 1:** Published data on fermentation of macroalgae by LAB and/or yeast aiming at the production of fermented seaweed food and feed products.

Macroalgae	Inoculum	Scale and Operation Mode	LA Titer	Other Fermentation Products	Fermentation Purpose	Reference
*Laminaria digitata* (Phaeophyceae)	*Lacticaseibacillus rhamnosus* (formerly *Lactobacillus rhamnosus*)	100 mL flasks; (24 h)	1.0 g/L	Acetic acid (0.06 g/L) *L. rhamnosus* (5.0 × 10^9^ CFU/mL)	Seaweed with higher antioxidant activity for probiotic product development	Gupta et al., 2012 [22]
*Saccharina latissima * (formerly *Laminaria japonica*) (Phaeophyceae)		100 mL flasks; (16h)	0.9 g/L	Acetic acid (0.7 g/L) *L. rhamnosus* (2.5 × 10^9^ CFU/mL)		
*Saccharina latissima*	*Lactobacillus plantarum*	Vacuum-packed sterile bags 20 × 30 cm (90 days)	50–60 g/kg Dry Matter (DM)	Acetic acid (3.7 g/L)	Ruminant feed ingredient	Campbell et al., 2020 [23]
*Fucus vesiculosos* (Phaeophyceae)			<5 g/kg DM	Acetic acid (3.1 g/L)		
*Pyropia yezoensis* (Rhodophyta)	*Terribacillus* *halophilus*	Plastic bags; (2 years)	0.6 g/L	Peptides (14.9 g/L) Folic acid (0.78 mg/kg) Taurine (6.17 mg/kg) vitamin B12 (0.14 mg/kg)	High-salt seaweed sauce as a novel nutritional product	Uchida et al., 2017 [24]
*Ulva reticulata* (Ulvophyceae)	*L. plantarum* *S. cerevisiae*	10 L fermenter (48 h)	7.6 g/L	*L. plantarum * (3.0 × 10^8^ CFU/mL)	Silage preparation for prawn larval development	Felix and Pradeepa, 2011 [25]
				*S. cerevisiae* (5.0 × 10^8^ CFU/mL)		
*Saccharina latissima* (formerly *Laminaria japonica*)	*L. plantarum* *S. cerevisiae*	Shake flasks (48 h)	na	*L. plantarum * (1 × 10^8.9^ CFU/mL) *S. cerevisiae * (1 × 10^7.5^ CFU/mL)	Hypolipidemic action	Qiulin Yue et al., 2021 [26]
*Undaria pinnatifida * (Phaeophyceae)	*Levilactobacillus brevis* (formerly *Lactobacillus brevis*); *Debaryomyces hansenii* (formerly *Saccharomyces hansenii*) and *Candida* sp.	Sterile 10-L polycarbonate bottles (18 months)	5.4 g/L	na	Marine silage preparation for young pearl oysters	Uchida et al., 2004 [27]

Legend: LA—lactic acid; *L. plantarum*—*Lactobacillus plantarum*; *L. rhamnosus*—*Lacticaseibacillus rhamnosus*; *L. casei*—*Lactobacillus casei*; *L. brevis*—*Levilactobacillus brevis*; *D. hansenii*—*Debaryomyces hansenii*; *T. halophilus*—*Terribacillus halophilus*; na—not available.

**Table 2:** Proximate composition of unprocessed and fermented *Ulva*. All fermented products (freeze-dried broths) were obtained through fed-batch fermentation of 813 mL/L seaweed slurry (pre-treated with 3% H_2_SO_4_), supplemented with 40 mL/L CSL, 2 g/L di-ammonium hydrogen citrate, 0.2 g/L MgSO_4_, and 0.05 g/L MnSO_4_. F1 stands for the 4LAB consortium fermentation broth, F2 for the yeast and 4LAB co-fermentation broth, and F3 for the yeast fermentation broth. F1 and F2 were derived from *Ulva* batch 1 and F3 from *Ulva* batch 2. Values are expressed as mean ± standard deviation. Values below quantification limits are indicated with (-).

Parameter	Units	*Ulva* Batch 1	*Ulva* Batch 2	F1: 4LAB Fermentation	F2: Yeast + 4LAB Fermentation	F3: Yeast Fermentation
Total Solids	%	82.5 ± 0.5	79.3 ± 2.2	89.7 ± 1.8	88.9 ± 0.5	84.0 ± 0.9
Moisture	%	17.5 ± 0.5	20.7 ± 2.2	10.3 ± 1.8	11.1 ± 0.4	16.0 ± 0.9
Ashes	% dw	26.0 ± 1.0	21.0 ± 1.8	15.0 ± 0.8	10.0 ± 2.2	42.0 ± 1.4
Total sugars	% dw	43.4 ± 0.6	50.2 ± 0.5	3.2 ± 0.8	2.7 ± 0.5	24.7 ± 0.4
Glucose	% dw	31.0 ± 1.0	29.6 ± 3.1	3.2 ± 0.8	2.7 ± 0.5	8.0 ± 0.9
Xylose	% dw	3.6 ± 0.8	9.3 ± 0.3	-	-	7.0 ± 0.1
Rhamnose	% dw	8.9 ± 3.2	11.3 ± 0.2	-	-	9.7 ± 0.3
Lactic Acid	% dw	-	-	42.1 ± 0.6	43.2 ± 0.3	-
Protein	% dw	15.2 ± 0.3	5.4 ± 0.5	16.4 ± 0.0	19.9 ± 0.3	23.5 ± 0.0

**Table 3:** Measured antioxidant (DPPH, ABTS radical scavenging, and reducing power), and chelating activities (Cu^2+^ and Fe^2+^) of freeze-dried broths from *U. rigida* fed-batch fermentations. EC_50_ values express the concentration of broth sample (mg/mL) needed to decrease by half the concentration of radical/ion in solution, while the reducing power refers to the sample concentration required to achieve an absorbance of 0.5 in the test.

Sample	ABTS EC_50_ (mg/mL)	DPPH EC_50_ (mg/mL)	Reducing Power Abs = 0.5 (mg/mL)	QCu EC_50_ (mg/mL)	QFe EC_50_ (mg/mL)
F1: 4LAB	16.5 ± 0.68	8.42 ± 0.03	5.08 ± 0.17	3.46 ± 0.12	-
F2: 4LAB + Yeast	10.1 ± 0.77	6.02 ± 0.26	3.81 ± 0.00	3.01 ± 0.02	-
F3: Yeast	10.4 ± 0.52	9.22 ± 0.04	3.10 ± 0.03	2.29 ± 0.09	11.01 ± 0.51

**Table 4:** Yield of hydrolysis (%) of *Ulva* polysaccharides calculated as the ratio between the total released sugars (TRS) value after each treatment scheme and the TRS value after total hydrolysis by the NREL protocol.

Saccharification Treatment	Acid Concentration (% *w*/*v*)				
	0.0	0.5	1.0	3.0	5.0
H_2_SO_4_	1	2	12	42	53
H_2_SO_4_ + enzymatic hydrolysis	n.d	n.d.	49	56	n.d.
HCl	2	7	26	54	46

n.d.: not determined.

**Table 5:** Yields of lactic acid and ethanol on total sugar consumption (g/g) in the shake flask cultivation assays on *Ulva* hydrolysates, with the 4LAB consortium and with *S. cerevisiae*, respectively.

Assay	1% (*w*/*v*) H_2_SO_4_ Acid Hydrolysis + Enzymatic Hydrolysis	3% (*w*/*v*) H_2_SO_4_ Acid Hydrolysis	5% (*w*/*v*) H_2_SO_4_ Acid Hydrolysis
4LAB cultivation	0.83	1.24	0.54
Yeast cultivation	0.31	0.25	0.18

## Data Availability

Data are contained within the article.
